# KDM4B-mediated reduction of H3K9me3 and H3K36me3 levels improves somatic cell reprogramming into pluripotency

**DOI:** 10.1038/s41598-017-06569-2

**Published:** 2017-08-08

**Authors:** Jingwei Wei, Jisha Antony, Fanli Meng, Paul MacLean, Rebekah Rhind, Götz Laible, Björn Oback

**Affiliations:** 1 AgResearch Ruakura Research Centre, Hamilton, New Zealand; 2 Animal Science Institute, Guangxi University, Nanning, P.R. China; 3 University of Otago, Department of Pathology, Dunedin, 9016 New Zealand

## Abstract

Correct reprogramming of epigenetic marks is essential for somatic cells to regain pluripotency. Repressive histone (H) lysine (K) methylation marks are known to be stable and difficult to reprogram. In this study, we generated transgenic mice and mouse embryonic fibroblasts (MEFs) for the inducible expression of KDM4B, a demethylase that removes H3 K9 and H3K36 trimethylation (me3) marks (H3K9/36me3). Upon inducing *Kdm4b*, H3K9/36me3 levels significantly decreased compared to non-induced controls. Concurrently, H3K9me1 levels significantly increased, while H3K9me2 and H3K27me3 remained unchanged. The global transcriptional impact of *Kdm4b*-mediated reduction in H3K9/36me3 levels was examined by comparative microarray analysis and mRNA-sequencing of three independent transgenic MEF lines. We identified several commonly up-regulated targets, including the heterochromatin-associated zinc finger protein 37 and full-length endogenous retrovirus repeat elements. Following optimized zona-free somatic nuclear transfer, reduced H3K9/36me3 levels were restored within hours. Nevertheless, hypo-methylated *Kdm4b* MEF donors reprogrammed six-fold better into cloned blastocysts than non-induced donors. They also reprogrammed nine-fold better into induced pluripotent stem cells that gave rise to teratomas and chimeras. In summary, we firmly established H3K9/36me3 as a major roadblock to somatic cell reprogramming and identified transcriptional targets of derestricted chromatin that could contribute towards improving this process in mouse.

## Introduction

Different somatic cell types within an individual are morphologically and functionally diverse, yet they share the same genetic information. Epigenetic modifications, such as DNA methylation and histone modifications, control cell-specific gene activity and stabilize phenotypic differences. ‘Reprogramming’ of these epigenetic marks occurs twice in normal mammalian development, first during gametogenesis and next during preimplantation embryogenesis^1^. This second wave resolves the early parental asymmetry in histone modifications, DNA methylation and chromatin proteins to allow for correct embryonic gene activation^2, 3^. It generates methylation marks in both DNA and histones that coincide with the first differentiation event during development, namely the specification of outer trophoblast and inner cell mass (ICM) of the early blastocyst^4^. The ICM later segregates into hypoblast and epiblast that develop into extra-embryonic and embryo tissues, respectively. Early epiblast cells are naturally pluripotent, i.e. capable of engendering all somatic and germ cell types in an adult animal, but lose this ability as development proceeds^5^. However, their transient pluripotency can be permanently captured in embryonic stem cell (ESC) lines. Taken together, these two reprogramming periods restore cellular potency to a pluripotent ground state^5^.

Radical manipulations, such as induced pluripotent stem cell (iPSC) derivation or somatic cell nuclear transfer (SCNT) cloning can artificially reprogram somatic cells^6^. Derivation of iPSCs requires ectopic expression of defined pluripotency factors, resulting in cells that are highly similar to ESCs in morphology, gene expression and germline competence^7^. During SCNT, a somatic cell nucleus is transplanted into an enucleated oocyte where its epigenetic marks are cleared by ill-defined reprogramming factors. In some cases it regains totipotency, i.e. the ability to form all embryonic and extra-embryonic lineages in a viable animal. Compromised development of the NT embryo is associated with aberrant methylation patterns of DNA^8^ and histones^9^ and dysregulation of gene expression^10^. Several pharmacological chromatin modifiers have been implicated in facilitating epigenetic reprogramming^11^. These drugs induce hyperacetylated, transcriptionally permissive chromatin and can improve iPS colony formation^12^ as well as cloning efficiency to term in mouse^11^ and pig^13^. Despite these advances, the reprogramming efficiency of somatic cells remains low due to a strong resistance to erase the epigenetic memory of previous lineage decisions and restart embryonic gene transcription^14^.

Pluripotent chromatin is characterised by an open configuration which extends into constitutive heterochromatin domains, such as pericentromeric satellite repeats, creating a transcriptionally permissive genome^15–19^. Histone (H) lysine (K) methylation (me) marks play a key role in this context. Consequently, several marks, including H3K4, -K9, -K27, -K79 and H4K20, have been associated with restricting reprogrammability of the genome. For example, pharmacological inhibition of histone deacetylation and genetic depletion of H3K9 methyltransferases force heterochromatin decompaction, increasing iPSC reprogramming efficiency^12, 20, 21^. Overexpressing histone lysine demethylases (KDMs), which reduce transcriptionally activating H3K36 and H3K4 modifications, increase efficiency of iPSC reprogramming^22, 23^. Particularly H3K9 marks, which persist through multiple cell divisions^24, 25^ pose a critical epigenetic barrier in cell reprogramming. We showed that by overexpressing KDM4B, H3K9me3 was demethylated and ESC reprogramming into cloned mouse embryos was improved^26^. A subsequent study confirmed that providing SCNT embryos with exogenous *Kdm4d*, which only targets H3K9me3, or depleting histone lysine methyltransferases (KMTs) in donor cells markedly increased clone development *in vitro* and *in vivo* ^27^. By contrast, accumulation of compact heterochromatin domains disrupts ESC self-renewal and alters differentiation potential^15, 28^. Reprogramming into iPSCs is also promoted by *Kdm3a* overexpression^29^ and genetic H3K9 KMT depletion^20, 21, 29–31^, possibly by restricting initial binding and expression of pluripotency factors located in heterochromatic regions^20^. Collectively, this suggest that heterochromatin obstructs re-establishing of pluripotency.

Here we investigated the consequences of *Kdm4b* overexpression on somatic H3K9me3 and H3K36me3 (H3K9/36me3) levels and gene expression in mouse embryonic fibroblasts (MEFs). We show a strong decrease in H3K9/36me3, which was accompanied by changed expression levels of 15 genes, including up-regulation of the heterochromatin-associated zinc finger protein 37 (*Zfp37*). Reduced H3K9me3 and H3K36me3 levels strongly increased reprogramming efficiency into cloned embryos and iPSCs, beyond what has been observed with embryonic donors.

## Results

### Dox-induced expression of Kdm4b-EGFP

**Figure 1:**
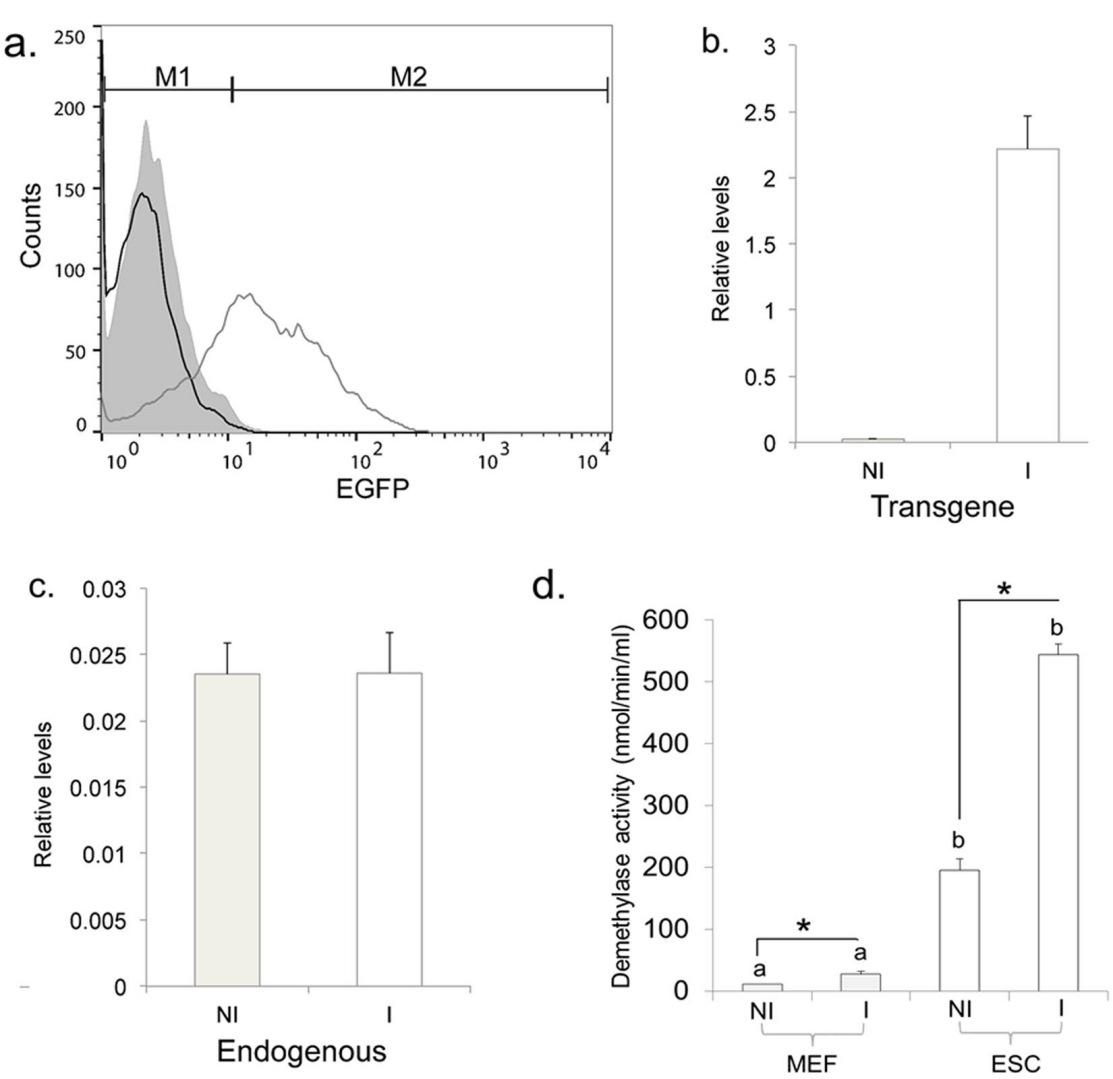
Characterization of inducible *Kdm4b*-EGFP expression. (**a**) Flow cytometry analyses of KDM4B-EGFP induction. Green fluorescence was determined using the FL1 EGFP emission channel. The range of intensities for green fluorescent cells (M2) is indicated. Relative cell number counts are plotted as a function of variable intensities of green fluorescence from individual cells. Black line graph: non-induced cells; grey line graph: induced cells; grey filled graph: induced cells 1 day after removal of doxycycline. (**b**,**c**) Kdm4b expression levels. Shown are the relative expression levels of transgene-derived Kdm4b (**b**) and endogenous Kdm4b (**c**) compared to the geomean of four housekeeping genes (n = 3 replicates). Results for non-induced (NI) and induced (I) *F-Kdm4b* MEFs are represented by grey and white bars ± SEM. (**d**) Demethylase activity in *F-Kdm4b* MEFs and ESCs. Results for non-induced (NI) and induced (I) *F-Kdm4b* MEFs and ESCs are represented by grey and white bars ± SEM, respectively (n = 3 replicates); Bars with an asterisk (*) differ P < 0.05 between treatments (NI vs I); bars with different letters (**a,b**) differ P < 0.05 between cell types (MEF vs ESC) of the same treatment by two-tailed unpaired t-tests.

MEFs were engineered for doxycycline (Dox)-inducible expression of a functional (F) and an inactive mutated (M) form of *Kdm4b*, with both transgenes fused to EGFP^26^. Selected *F-Kdm4b* MEF lines were Dox-induced for two days and expression of the *Kdm4b-EGFP* transgene analyzed by flow cytometry. Up to 82% (71 ± 2%, n = 24 replicates across six different cell lines) of *F-Kdm4b* cells displayed EGFP fluorescence (Fig. 1a). Within 24 hours after Dox removal, cells returned to a profile that was indistinguishable from non-induced cells, confirming reversibility of inducible transgene expression (Fig. 1a). Compared to non-induced controls, there was a 97-fold increase in transgene-derived *F-Kdm4b* expression (Fig. 1b). This did not affect endogenous *Kdm4b* mRNA levels (Fig. 1c). Global activity for demethylases containing the catalytic JMJC domain increased 2.5- and 2.8-fold in Dox-induced *F-Kdm4B* MEFs and ESCs, respectively (Fig. 1d). Overall, JMJC family demethylase activity was ~20-fold higher in ESCs than in MEFs (Fig. 1d).

### Induced F-KDM4B specifically reduces H3K9/36me3 in MEFs

**Figure 2:**
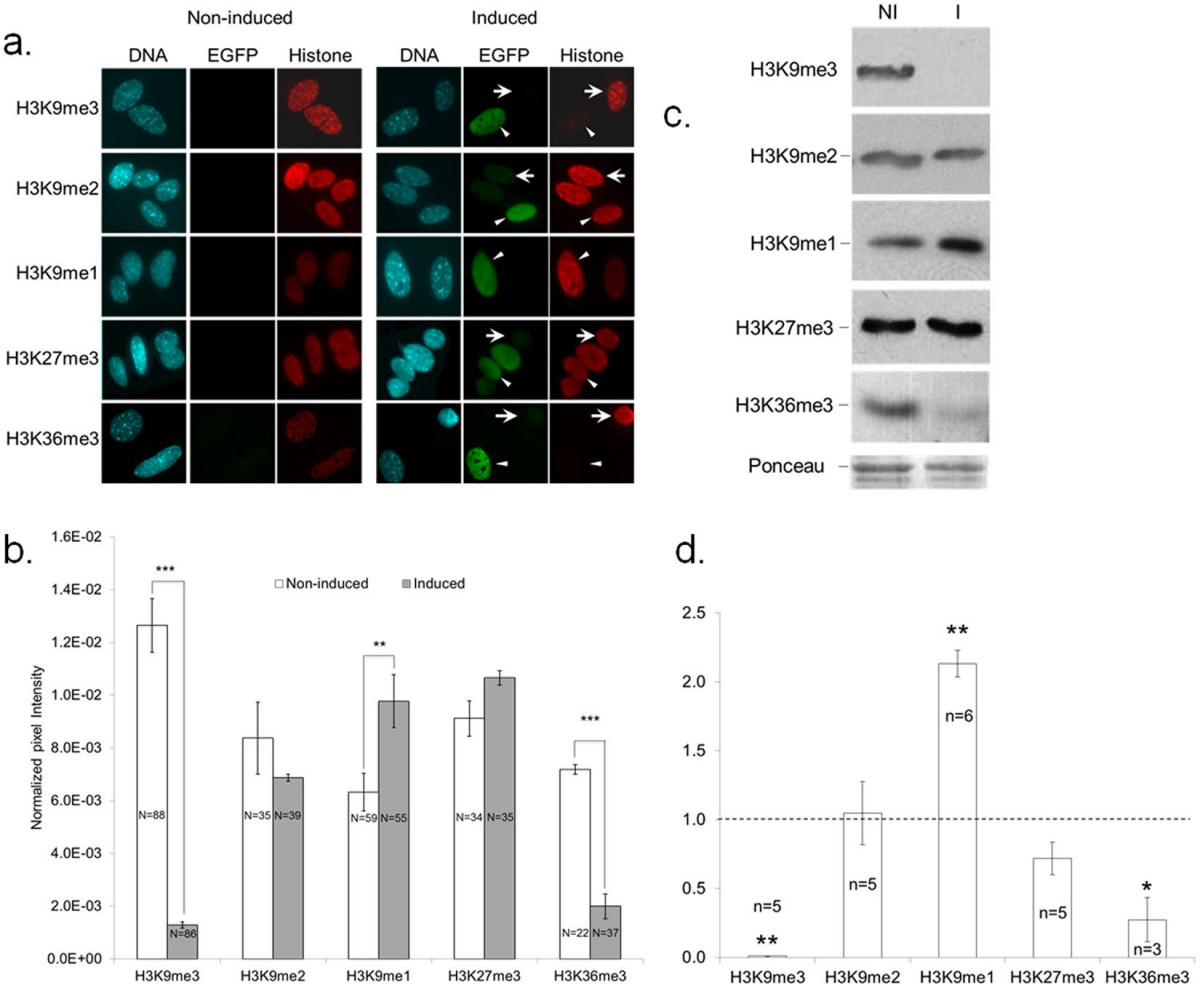
Characterization of histone methylation in *Kdm4b*-EGFP expressing MEFs. (**a**) Immunofluorescence of histone modifications in non-induced (NI) vs induced (I) MEFs. Cells were co-stained for EGFP and histone modification antibodies. Arrowheads and arrows indicate EGFP-positive and –negative nuclei, respectively. (**b**) Quantification of global histone methylation in NI vs I MEFs. Values represent normalized pixel intensity ± SEM. Asterisks indicate significant differences. N = number of nuclei quantified, RU = relative units; ***P < 0.001; **P < 0.01. (**c**,**d**) *Kdm4b*-dependent changes in levels of H3K9 methylation states. (**c**) Histone extracts from NI vs I MEFs were quantified for the indicated histone modifications by western blot. Shown are cropped representative blots for the different methyl states of H3 and a cropped representative Ponceau staining. (**d**) Quantification of western analysis. Results represent H3 methylation levels in induced cells as a percentage of non-induced levels ± SEM. Dotted line indicates NI level; n = number of replicate experiments, Bars with an asterisk differ from NI level at **P < 0.005, *P < 0.05, as determined by two–tailed paired t-test on normalised band intensities.

To determine the effect on histone methylation, induced and non-induced *F-Kdm4b* MEFs were analysed for H3K9me1/2/3, H3K36me3 and H3K27me3 levels by semi-quantitative immunofluorescence (Fig. 2a). In F-KDM4B-EGFP positive nuclei, signal intensity for H3K9me3 and H3K36me3 was 10-fold and 4-fold lower, respectively, than in non-induced controls, while H3K9me1 levels were 1.5-fold increased (Fig. 2b). No changes were apparent for H3K9me2 and H3K27me3. To independently validate these results, we performed western blot analyses on bulk histones (Fig. 2c). Histones were extracted from two cell lines with an average induction of 70 ± 6%. Upon induction of *F-Kdm4b*, H3K9me3 and H3K36me3 levels dropped ~100-fold and ~5-fold, respectively, compared to non-induced controls, accompanied by a ~2-fold increase in H3K9me1 levels (Fig. 2d). H3K9me2 and H3K27me3 levels were not significantly altered.

We also tested whether H3K9 acetylation (H3K9ac) changed upon H3K9/36me3 reduction. Following induction for 48 h, H3K9ac levels were not affected as determined by immunofluorescence (Fig. S1a) and quantification of pixel intensities (Fig. S1b). Likewise, H4K12ac and H4K16ac were not significantly changed after 48 h induction (data not shown).

### Gene expression profiling

**Figure 3:**
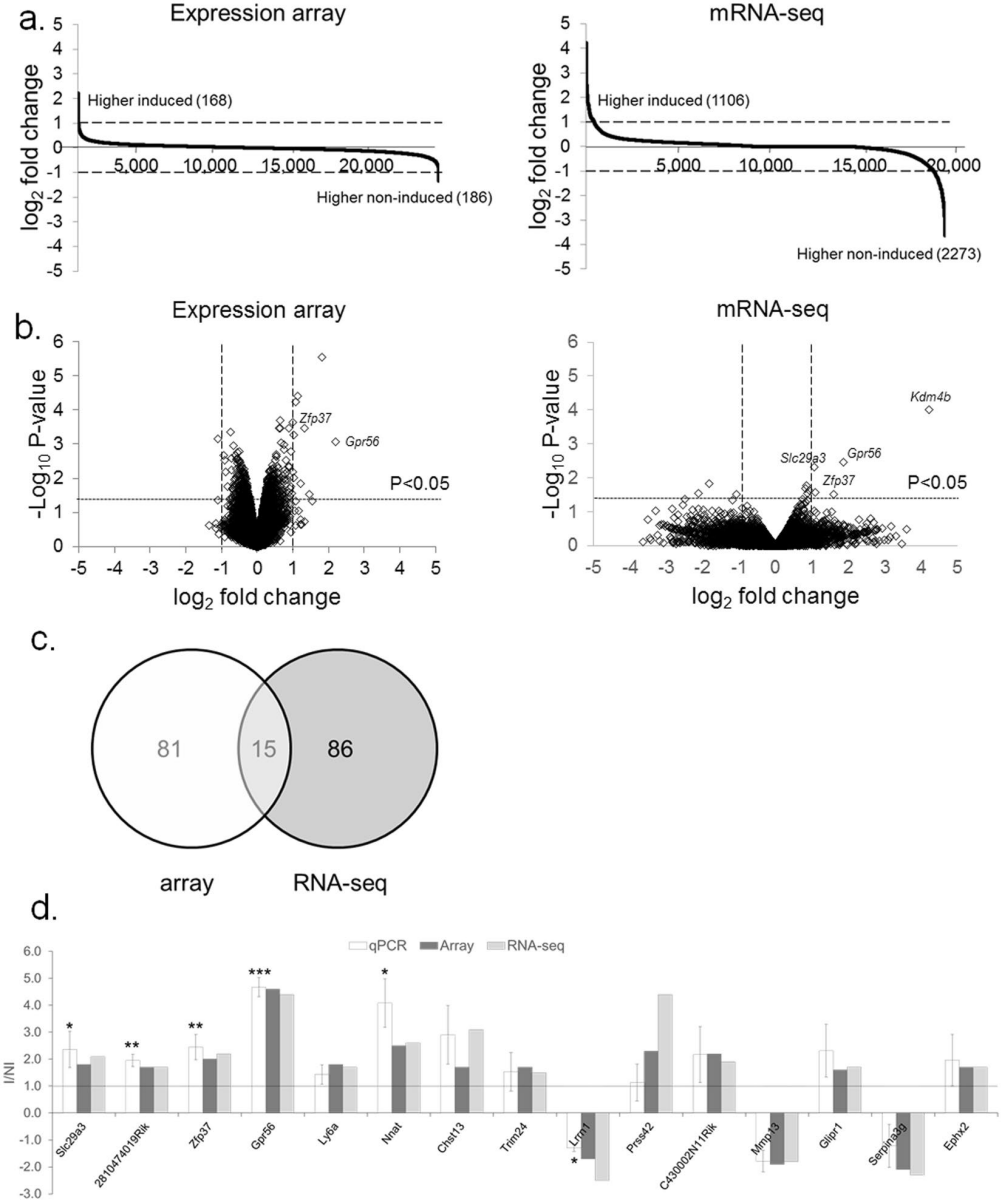
Transcriptome profiling of *Kdm4b* MEFs. (**a**) Fold change (log2 values) in transcript level of all genes under non-induced (NI) vs induced (I) conditions as measured by expression array and mRNA-seq. Gene expression levels of three MEF lines were averaged, after which ratios were calculated. A two-fold change is indicated by the stippled horizontal line. (**b**) Volcano plots of gene expression changes. Fold-changes were normalized on the NI control. A two-fold change is indicated by the stippled vertical lines. A dotted horizontal line indicates P = 0.05 from two-tailed t-test. (**c**) Number of genes that are significantly deregulated (1.5-fold, P < 0.05) in the same direction in microarray vs mRNA-seq. (**d**) qPCR validation of candidate genes. Shown are the ratios of I over NI relative expression levels, normalized on the geomean of three housekeeping genes. Dotted horizontal line indicates NI level. Error bars: standard error of ratios determined from 3–6 biological repeats per gene. Bars with an asterisk differ between NI vs I at *P < 0.05; **P < 0.005; ***p < 0.001 as determined by two–tailed t-test on mean expression levels.

The global transcriptional impact of *Kdm4b*-mediated reduction in H3K9/36me3 levels was examined by microarray, mRNA-seq and qPCR. Three independent female *F-Kdm4b* MEF lines, either induced or non-induced, were used for all gene expression studies. On the microarray, a total of 137,489 probes, representing 24,203 different genes, were detected (Table S1). In total, 599 were significantly altered (Fig. 3b, Table S1). From these, 168 (63 with P < 0.05) genes had a more than 1.5-fold higher and 186 (33 with P < 0.05) a 1.5-fold lower transcript abundance when *Kdm4b* was induced (Fig. 3a). The maximal difference was a 4.6-fold up- and 2.1-fold down-regulation (Table S1). We also performed microarray analyses of *F-Kdm4b* ESC clones. In total, 6012 were significantly altered (Table S1). From the same 24,203 genes as on the MEF array, 1467 (540 with P < 0.05) genes had a more than 1.5-fold more and 616 (568 with P < 0.05) a 1.5-fold less transcript when *Kdm4b* was induced, with a maximal 4.8- and 6.2-fold up- and down-regulation, respectively (Table S1). Four array probes were found to match the *Kdm4b* gene itself. However, none of these covered amino acids 1–424, corresponding to the overexpressed truncated version of *Kdm4b*. This explains why *Kdm4b* overexpression was not detected on the arrays. From the same MEF RNA that was used for microarrays, mRNA was isolated for sequencing. Across all six samples, an average of 83 megabases were read with a Q30 > 91.9 and a mean quality score per base of 35.2. These represented 19,478 different genes. From these, 104 genes were significantly altered (Fig. 3b). Out of the total, 1106 (35 with P < 0.05) genes had a more than 1.5-fold higher and 2273 (66 with P < 0.05) a 1.5-fold lower transcript abundance when *Kdm4b* was overexpressed (Fig. 3a). The maximal difference was a 26-fold up- and 31-fold down-regulation (Table S1). The *Kdm4b* gene itself was 15-fold overexpressed (P < 5E-09). Comparing microarray and mRNA-seq results, we found 16,061 genes to be present in both data sets. From these, 15 genes were deregulated in the same direction, using a 1.5-fold change and P < 0.05 as cut-offs (Fig. 3c, Table 1). This overlap was significant (P < 0.05). Taking into account the ESC expression array, there were four additional 1.5-fold up-regulated genes (*Ly6a, Ly6c2, Ly6f*, *Sprr2a3*) but only one (*Ly6a*) was significant between all array (MEF, ESC) and mRNA-seq (MEF) data sets (Table S1). All of these were confirmed to change their expression in the same direction by qPCR validation, with 12 being up- and 3 being down-regulated (Fig. 3d). This shows a 100% concordance between microarray, mRNA-seq and qPCR data, which was significant for 40% of candidates (*Zfp37, 2810474O19Rik, Gpr56, Slc29a3, Nnat* and *Lrrn1*). Overall, the fold-changes in expression levels of all five genes were remarkably similar between microarray, mRNA-seq and qPCR data (Table 1).

**Table 1:** Differentially expressed genes in *Kdm4b*-overexpressing MEFs.

Gene symbol	Gene name	Chr.	Array		mRNA-seq		qPCR	
			n-fold	P	n-fold	P	n-fold	P
*Zfp37*	Zinc finger protein 37	7	2	0.0006	2.2	0.0004	2.4	0.0066
*Trim24*	Tripartite motif-containing 24	X	1.7	0.01139	1.5	0.0306	1.5	0.7523
*Slc29a3*	Solute carrier 29, member 3	3	1.8	0.00034	2.1	0.0001	2.4	0.0214
*Serpina3g*	Serine peptidase inhibitor A 3 g	12	−2.1	0.00071	−2.3	0.0058	−1.2	0.7241
*Prss42*	Protease, serine 42	12	2.3	0.01417	4.4	0.0096	1.1	0.9026
*Nnat*	Neuronatin	7	2.5	0.00035	2.6	0.0003	4.1	0.0351
*Mmp13*	matrix metallopeptidase 13	14	−1.9	0.00208	−1.8	0.0187	−1.8	0.5326
*Ly6a*	Lymphocyte antigen 6 complex, locus A	15	1.8	0.00291	1.7	0.0342	1.4	0.1145
*Lrrn1*	Leucine rich repeat protein 1	10	−1.7	0.00044	−2.5	0.0364	−1.3	0.0282
*Gpr56*	Adhesion G protein-coupled receptor G1	17	4.6	0.00083	4.4	0.0019	4.7	0.0000
*Glipr1*	GLI pathogenesis-related 1 (glioma)	11	1.6	0.04462	1.7	0.0252	2.3	0.5005
*Ephx2*	Epoxide hydrolase 2	8	1.7	0.00996	1.7	0.0488	2.0	0.2124
*Chst13*	Carbohydrate sulfotransferase 13	X	1.7	0.00105	3.1	0.0094	2.9	0.6446
*C430002N11Rik*	—	5	2.2	0.00004	1.9	0.0146	2.2	0.9877
*2810474O19Rik*	—	2	1.7	0.00746	1.7	0.0009	2.0	0.0036

Chr. = chromosome, ND = not determined.

Using poly(A)-selected mRNA-seq in *Kdm4b* MEFs, altered transcription was also evident for many repeat regions, including satellite sequences, long and short interspersed nuclear elements (LINEs, SINEs), DNA transposons and long terminal repeat elements containing endogenous retroviruses (ERVs) (Table S2). We observed mostly a down-regulation of satellite sequences (59%), LINEs (84%), SINEs (83%), DNA transposons (74%) and ERVs (69%). This was also true for subsets of LINEs, for example, of the L1Md_A family (80% down-regulated). Full-length (>6 kb) ERV type transposons, on the other hand, showed the opposite trend and were 100% up-regulated.

Functional annotation clustering of differently expressed (DE) genes by Gene Ontology (GO) and QIAGEN’s Ingenuity® Pathway Analysis (IPA®, QIAGEN Redwood City) revealed DE genes as enriched for canonical pathways associated with pluripotency in ESCs, including oncostatin M signaling^32^ and transcriptional regulation of pluripotency (Table S3). Cell compromise and maintenance were the main functional categories, with cell cycle as the top associated network (Table S3).

### Reduced H3K9/36me3 levels are restored after nuclear transfer

**Figure 4:**
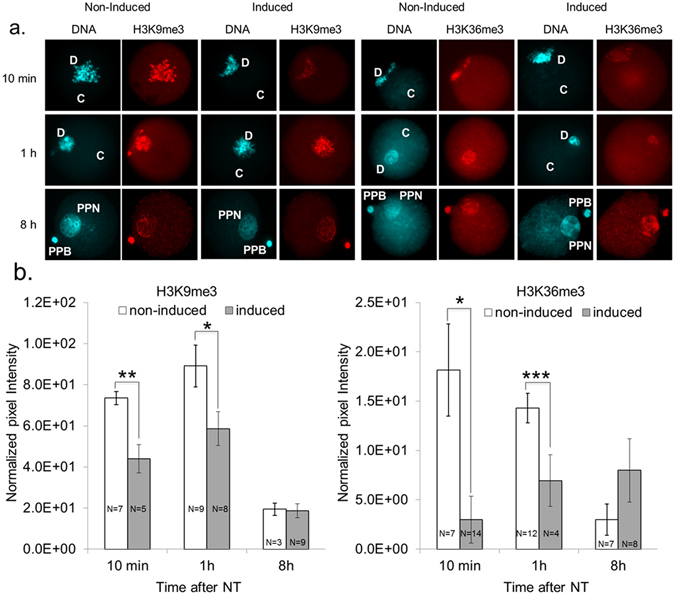
H3K9/36me3 levels in NT reconstructs generated from induced vs non-induced *Kdm4b* MEFs. (**a**) NT reconstructs were fixed and co-stained for DNA (H33342) and antibodies specific for histone modifications. Cells were analyzed 10 minutes (upper), 1 h (middle) and 8 h (lower panel) after transfer of donor DNA (D) into cytoplast (C); One-cell reconstructs were fixed and stained with H33342 (DNA) and antibodies specific for H3K9me3 and H3K36me3. PPB = pseudo polar body, PPN = pseudo-pronucleus. (**b**) Quantification of immunofluorescence analysis in (**a**). Values represent normalized pixel intensity ± SEM. N = number of NT reconstructs quantified. Different letters indicate significant differences between MEFs from the same groups (induced or non-induced) at different time points, ***P < 0.0001, **P < 0.01, *P < 0.05 by two-tailed unpaired t-tests.

We first adapted our zona-free NT cloning procedure from ESCs to MEFs. MEFs were incubated in pronase prior to lectin-sticking and couplet formation^33^. This pre-treatment tripled mitotic MEF fusion efficiency from 57/306 = 19% (n = 7) to 250/400 = 63% (n = 6), using otherwise standard conditions (P < 0.0001). We then examined how the reduced H3K9/36me3 levels from induced *F-Kdm4b* donors changed after NT and subsequent *in vitro* embryo development under non-induced conditions. NT reconstructs from induced and non-induced *F-Kdm4b* MEFs were fixed at various time points after NT and analyzed by immunofluorescence for the presence of H3K9/36me3 (Fig. 4a). Within ten minutes after fusing MEFs with the cytoplast, the EGFP signal became undetectable in the NT reconstruct and remained so even after formation of a single pseudo-pronucleus. Reduced H3K9/36me3 signals were still observed in NT reconstructs from induced compared to non-induced donors ten minutes and one hour after fusion (Fig. 4b). At eight hours post fusion, both the newly formed pseudo-pronucleus and extruded pseudo-polar body displayed similar H3K9/36me3 intensities in NT reconstructs derived from induced and non-induced donor cells (Fig. 4b). Thus the initially different H3K9/36me3 levels in MEFs were restored relatively rapidly.

### Reduced H3K9/36me3 levels improve in vitro development of cloned embryos

To evaluate whether MEFs with reduced H3K9/36me3 levels would improve donor cell reprogrammability, we determined their *in vitro* development. Culture was performed without Dox using induced vs non-induced *F-Kdm4b* cells and additional *M-Kdm4b* controls. Prior to each NT run, cells were validated for induction of *Kdm4b* by monitoring EGFP fluorescence. The average induction rate was 76 ± 8% (range 60–88%; n = 3). Reconstructs from induced *F-Kdm4b* MEFs developed similarly until the morula stage (induced vs. non-induced 9% vs. 7%) but 6-fold better into blastocysts (induced vs. non-induced 18% vs. 3% p < 0.0001) compared to non-induced donors (Table 2). By contrast, no significant differences were detected in control experiments using induced vs non-induced *M-Kdm4b* donor MEFs. A small number of *F-Kdm4b* embryos (N = 33) from different experiments (n = 4) were transferred into four recipients but none of them implanted.

**Table 2:** *In vitro* development of NT reconstructs.

Cell line	n	No. of NT reconstructs^a^	No. of morulae (% ± SEM)	No. of blastocysts (% ± SEM)	No. of morulae & blastocysts (% ± SEM)
*F-Kdm4b*, NI	8	182	13 (7 ± 2%)	6 (3 ± 2%)^c^	19 (10 ± 2%)^c^
*F-Kdm4b*, I	8	213	19 (9 ± 5%)	38 (18 ± 4%)^d^	57 (27 ± 4%)^d^
*M-Kdm4b*, NI	4	125	7 (6 ± 2%)	5 (4 ± 2%)	12 (10 ± 2%)
*M-Kdm4b*, I	4	182	4 (2 ± 1%)	4 (2 ± 2%)	8 (4 ± 2%)

NI = non-induced, I = induced; ^a^all data relate to reconstructs that extruded a single polar body and were cultured until day 4; ^cd^Values within different superscripts within a column differ with P < 0.0001.

Treatment with different histone deacetylase inhibitors (HDACi) can significantly improve developmental efficiency of SCNT embryos^11^. To explore a possible relationship between HDACi and *Kdm4b*, we simultaneously treated *Kdm4d-*SCNT embryos with either trichostatin A (TSA) or scriptaid (SCR). These reagents improved SCNT morula and blastocyst rate ~4–15-fold, respectively, over non-induced controls (Table S4). However, neither reagent further increased development beyond the level already achieved by overexpressing *Kdm4b* (Table S4). Thus combining HDACi with *Kdm4b* had neither additive nor synergistic effects on reprogramming SCNT preimplantation stages, suggesting that they may exert their effects through a similar pathway.

### Improved reprogramming into iPSCs

**Figure 5:**
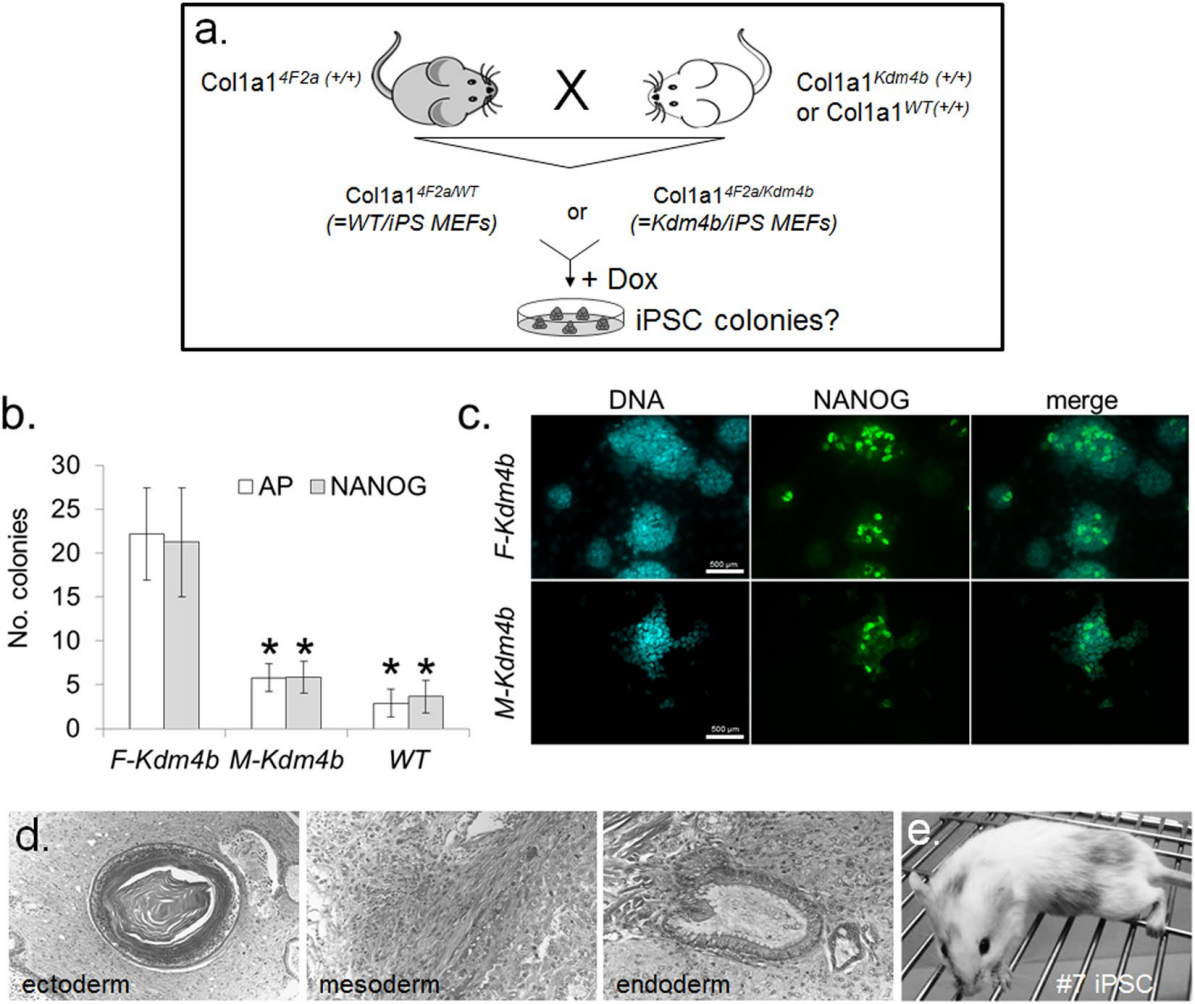
Derivation of iPSCs from reprogrammable *Kdm4b* and wild-type MEFs. (**a**) Breeding scheme to obtain reprogrammable MEFs for iPSC assay. (**b**) Formation of alkaline phosphatase (AP)- and NANOG-positive iPSC colonies from *F-Kdm4b*, *M-Kdm4b* and wild-type (WT) MEFs. Colony numbers ± SEM were counted following one week of Dox withdrawal after 21 days in culture; ^*^ *F-Kdm4b* differs from controls at P < 0.05 by two-tailed unpaired t-tests (n = 6 biological replicates). (**c**) Immunofluorescence against NANOG in primary colonies on D28, after one week of Dox withdrawal. (**d**) Histological sections of intramuscular teratomas from *Kdm4b* iPSC line #1, showing differentiation into all three germ layer derivatives. (**e**) Following blastocyst injection, iPSC line #7 contributes to coat-color chimera.

iPSC formation is a complementary cell reprogramming approach to SCNT (Fig. 5a). In both Col1a1^*4F2a/WT*^ (‘WT/iPS’) and Col1a1^*4F2a/F-Kdm4b*^ (‘*Kdm4b/iPS*’) MEFs, dome-shaped, tightly packed colonies with clear borders appeared around 12 days post-induction and one week following culture in iPSC medium^34, 35^. Upon Dox withdrawal after 12 days in culture, both MEF genotypes reverted to a fibroblast morphology and very few colonies remained. When Dox was removed nine days later, the number of stably induced, Dox-independent alkaline phosphatase- and NANOG-positive colonies was significantly higher in the *F-Kdm4b*-expressing compared to *M-Kdm4b* and wild-type MEFs (Fig. 5b). Non-induced MEFs of either genotype did not result in colony formation. Following repeated passaging without Dox (Fig. 5c), 4F2a/*F-Kdm4b*-derived iPSCs were assessed for pluripotency in functional assays. Following injection of three independent iPSC^*4F2a/F*-*Kdm4b*^ lines into SCID mice, large tissue masses, ranging from 10–20 mm in diameter, were harvested after seven weeks from five out of six hind legs (Table S5). Histological examination showed differentiation into ectoderm (squamous epithelium), endoderm (ciliated epithelium) and stromal mesoderm (Fig. 5d). These features were consistent with intramuscular grade 3 teratomas. To fully evaluate pluripotency, two iPSC^*4F2a/F*-*Kdm4b*^ lines were tested for their ability to contribute to chimeras. We obtained four live coat-colour chimeras (Table S6, Fig. 5e). Following repeated test matings, no germ line transmission was obtained from these chimeras.

## Discussion

Here we show that H3K9/36me3 poses major obstacles during somatic cell reprogramming and identify potential transcriptional targets of reduced H3K9/36me3 levels. We first generated mice that conditionally overexpress KDM4B. These provide a new animal model to study nuclear reorganization during cell differentiation, in particular the remodeling of heterochromatin architecture during developmental fate transitions^15, 19, 36, 37^. They also allow direct assessment of the stable, repressive H3K9me3 mark on nuclear reprogramming from various somatic cell types. Compared to ESCs, there was a greater decline of H3K9me3 (~63% vs ~99%) and greater increase of H3K9me1 (~130% vs ~213%) levels in *F-Kdm4b* MEFs^26^. This supports the notion that KDM4B-mediated demethylation converts H3K9me3 into H3K9me1. Apart from repressive H3K9me3, KDM4B also demethylates H3K36me2/3, which is mainly associated with transcriptional activation^38^. While its demethylase activity for this target is much weaker, decreasing H3K36me2 levels by only 10%^38^, we have found a 4-5 fold decrease in H3K36me3 levels.

How would reduced H3K9/36me3 make the donor genome easier to reprogram? H3K36me3 is present at transcribed genes, peaking toward the middle and 3′ ends of coding regions but mostly missing from promoters^39^. Interestingly, trimethylation of active H3K36 and repressive H3K27 do not coexist on the same H3 tail^39^. This broad mutually exclusive distribution of H3K36me3 over active euchromatin may prevent spreading and accumulation of silencing marks. Indeed, removal of H3K36me1/2/3 writers results in global loss of H3K36me3, redistributing H3K27me2/3 from its endogenous sites to active gene bodies and mis-regulating gene expression^39^. We may not have detected subtle changes in H3K27me3 abundance or staining pattern. For this, it might be worthwhile to interrogate regions enriched in H3K36me3 and H3K27me3, such as those involved in maintaining X-linked gene expression and X chromosome inactivation in females^40^, in particular when they have a negative impact on NT reprogramming^41^.

H3K9me3, on the other hand, is a repressive modification. It is recognized and bound by the heterochromatin protein HP1^42, 43^, causing heterochromatin formation^44^ and epigenetic silencing^45^. Therefore, H3K9me3-initiated heterochromatinization can restrict access to chromatin-binding factors and prevent activation of developmentally important genes. In addition, H3K9me3 may inhibit local deposition of activation marks, such as H3K9 acetylation, which we found globally unchanged. These changes in donor cell epigenotype altered transcription for 15 genes. Given that H3K9me3 generally marks gene-poor regions and accumulates over repetitive elements, such a relatively low number of differentially expressed genes is not surprising. Most genes were up-, not down-regulated, consistent with expectations from the published role of H3K9me3 as a transcriptional repressor. Whilst some megabase-scale H3K9me3 regions contain pluripotency genes (e.g. *Nanog, Sox2, Prdm14*), these candidates were not significantly effected in our screen. Among the changed transcripts, the only direct linkage to heterochromatin was *Zfp37*. The corresponding zinc finger protein is expressed in brain and testis, where it specifically associates with the heterochromatin adjoined to nucleoli^46^. It is not known what regulatory role ZFP37 plays in these tissues. Only one gene, *Ly6a* (or *Sca-1*), was up-regulated in both ESCs and MEFs. This gene is part of a large family of mostly GPI-linked cell-surface glycoproteins in lymphocytes. Monoclonal antisera against Ly6a are commonly used to identify hematopoietic stem cells but is also found on other leukocyte subsets^47^. The *in vivo* function of this protein remains largely unclear^47^. Overexpressing some of these candidate genes in donor cells or embryos may shed light on their function and directly improve cloned blastocyst formation.

Following NT, all donor transcripts and proteins will be transferred and ~1000-fold diluted in the cytosol of the recipient oocyte, where they could influence early embryonic gene expression. Numerous studies have shown that even though the donor cell genome is largely repressed by the late one-cell stage, some highly expressed donor transcripts remain detectable at both early and late mouse preimplantation stages^27, 48, 49^. It would be informative to measure the extent of this donor-specific gene and protein “memory” in *Kdm4b*-derived SCNT embryos.

In addition to the protein-coding and unannotated transcripts, H3K9me3-marked regions harbour repetitive sequences, which were also represented in our mRNA-seq data set. In ESCs, most H3K9me3 chromatin is targeted to repeat-rich intergenic and intronic regions and absent from coding sequences or regulatory elements, such as enhancers or promoters. The major repeat target regions for H3K9me3 outside constitutive heterochromatin are full-length (>6 kb) ERVs and LINEs^50^. This subpopulation of intact retrotransposons accumulates highest levels of H3K9me3, whereas the vast majority of degenerate and truncated ERVs and LINEs fail to be enriched for this mark. Following loss of H3K9me3 accumulation, the most significantly deregulated targets were major satellites, ERVs and intact LINEs, especially of the L1Md subfamily^50^. However, ERV and LINE-specific H3K9me3 enrichment and transcriptional dysregulation was not observed in immortalized MEFs^50^. Instead, genome-wide coverage of H3K9me3 in immortalized MEFs mainly resides in megabase domains that are not enriched for LINEs, ERVs, or other repeats but rather contain genes^20^. We observed increased transcriptional output from all full-length ERV (>6 kb) repeat regions. This may contribute to the better reprogramming of *Kdm4b*-expressing donor cells because activation of repeat sequences is important for preimplantation development^51, 52^. Our observation is consistent with the finding that *Kdm4d* partially relieves repression of murine ERV retrotransposons in 2-cell SCNT embryos^27^.

Globally reduced H3K9/36me3 levels in induced donor cells initially persisted in reconstructed embryos but returned to non-induced control levels within eight hours of NT. This is different from the prompt restoration of H3K9me3 marks previously observed for ESCs, which occurred within minutes of NT^26^. The difference is consistent with the greater H3K9me3 depletion in MEFs relative to ESCs, which may take longer to reprogram by histone methyltransferases active in the recipient cytoplasm. Indeed, RNA-seq and proteomics of metaphase-arrested oocytes has detected all known H3K9me3-specific KMT mRNAs and KMT1E protein, respectively^53^. This fast-acting cytosolic machinery would normally reprogram the incoming mouse sperm upon fertilisation, restoring H3K9 methylation in unmethylated male pronuclei within three hours of entry into the oocyte^54^. The rapid gain of methylation observed in MEF nuclei suggests fast enzymatic re-methylation of H3K9 sites rather than slow incorporation of already methylated histones. Concomitant with normalizing H3K9me3, there was a complete loss of KDM4B-EGFP signal in NT reconstructs, probably caused by rapid dissociation from the prematurely condensing donor chromatin and dilution in the oocyte cytoplasm^55^.

The apparent re-instatement of H3K9me3 levels was assessed by IF, which only provides a semi-quantitative read-out of global methylation levels. It is possible that some loci remained hypomethylated, for example the previously described reprogramming-resistant genomic regions in MEFs^27^. These heterochromatin regions are enriched for KMT1A/B-deposited H3K9me3 and low DNase I accessibility. Continued activation of locally H3K9me3-depleted regions may persist for some time, making the genes in these regions worthwhile candidates for further investigation of embryonic genome activation in *Kdm4b*-induced vs non-induced SCNT embryos. Thus even a very short-lived decrease in H3K9me3 levels could facilitate initial binding of oocyte reprogramming factors, triggering a ripple effect that persists after the global differences in H3K9me3 have disappeared.

In spite of their rapid apparent restoration, reduced H3K9me3 donor levels correlated with an almost tripled *in vitro* development of NT reconstructs. This is a ~5-fold greater increase than previously observed with ESCs using the same approach^26^, suggesting that a greater reduction in H3K9me3 levels is beneficial for NT-mediated reprogramming into blastocysts.

H3K9me3-mediated heterochromatin formation also acted as a reprogramming barrier for generating bona fide iPSCs. This is supported by several studies using both gain-of-function^30^ and loss-of-function^20, 21, 30^ approaches to reduce H3K9me3 to promote conversion of MEFs into iPSCs. The H3K9me3 barrier in mouse iPS reprogramming can be lowered by knocking down various H3 KMTs, primarily KMT1 variants^20, 21, 30^. Since iPSC generation is relatively slow compared to NT, extending over days and weeks, the immediate downstream gene networks necessary for reacquisition of pluripotency, are likely different. Accordingly, the only indispensable reprogramming factors for iPSC generation, Oct4/Pou5f1, has been shown to be dispensable in SCNT reprogramming^56^.

While we were able to generate cloned blastocysts with Kdm4b-EGFP MEFs, we did not obtain live offspring. Hence we cannot conclude that induction of *Kdm4b* in donor cells improved *in vivo* development of cloned embryos. Considering the substantial technical difficulties of cloning live mice, achieved by only a very small number of groups, our lack of success may be attributable to inherent technical difficulties. In another study, implantation and development to term was significantly higher in *Kdm4d*-injected than in control SCNT embryos^27^.

In summary, we demonstrate improved *in vitro* reprogramming into cloned embryos and iPSCs following *Kdm4b* overexpression in MEFs. It is plausible that targeted reduction of repressive H3K9me3 marks led to a derestricted genome with greater reprogrammability. Targeting other histone modification, such as H3K36me1/2 via KDM2 overexpression^22, 23^ and H3K79me via KMT4 knockout^57^, could further improve reprogramming efficiency. It remains to be seen if this cell-based epigenetic therapy is applicable to other mammalian species, such as, livestock or humans. In cattle, for example, serum starving donor cells reduces their histone methylation levels, which increased reprogramming into totipotency^58^. This provides a mandate to develop specific small molecule inhibitors (e.g. for KMT1) to improve epigenetic reprogramming without genetic intervention.

## Materials and Methods

### Transgenic *Kdm4b* mice

All methods were carried out in accordance with the New Zealand (NZ) Animal Welfare Act 1999 and approved by the Ruakura Animal Ethics Committee. To make germline chimeras, we used ESCs (C57/BL6 × 129SV) engineered for Doxycycline (Dox)-inducible expression of a functional (F) and an inactive mutated (M) form of *Kdm4b*, with both transgenes fused to EGFP^26^. Each transgene was integrated at the collagen 1 alpha 1 (Col1a1) locus and controlled by a tetO promoter, driven by the M2 reverse tetracycline transactivator (*M2-rtTA*) in the *Rosa26* locus^26^. Chimeras and offspring were PCR-screened for *rtTA* and *Kdm4b-EGFP* transgenes and absence of the wild-type loci (Table S7).

### Cell culture and transgene induction

Homozygous animals (R26^*rtTA*+/+^, Col1a1^*Kdm4b*+/+^) were mated and mouse embryonic fibroblasts (MEFs) derived from day 13.5 embryos (Table S8). MEFs were cultured in DMEM/F12 with 10% fetal bovine serum (FBS) and 1x MEM non-essential amino acids. ESCs were cultured feeder-free on 0.1% gelatine in DMEM/F12 with 20% FBS, 100 µM β-mercaptoethanol, 1x MEM non-essential amino acids, 2000 U/ml of LIF and 0.4 µM PD0325901. MEFs were analyzed for EGFP fluorescence on a FACSCalibur™ flow cytometer.

### RNA isolation and cDNA synthesis

RNA was isolated using the RNeasy mini kit (Qiagen). Genomic DNA was removed through digestion with DNase 1 for 1 h at 37 °C, followed by heat-inactivation for 10 min at 65 °C. cDNA was synthesized with SuperScript III First-Strand Synthesis SuperMix using oligo-(dT) primers.

### Quantitative PCR

Target sequences (Table S6) were amplified using the Syber Premix ExTaq (Takara) with a Rotorgene 6000 (Corbett) or LightCycler^®^ 2.0 (Roche). Comparative quantification in the Rotorgene Software was used to determine amplification efficiency of each reaction^59^. Transcript levels were determined relative to the geometric mean of four housekeeping genes (*Gapdh*, *Actb*, *Hprt*, *Gusb*) while normalizing for amplification efficiency^60^. Reverse transcriptase was omitted in one sample for each cDNA synthesis reaction (“-RT” control). Overexpression plasmids served as positive controls and water as negative control (“no template”). Each sample was run in technical triplicates. Product identity was confirmed by melting curve analysis.

### Microarray and mRNA-seq sample preparation

One *F-Kdm4b* ESC clone^26^, in biological triplicates, and three female *F-Kdm4b* MEF lines were used for global transcript profiling. A sub-sample from each line showed >70% induction by flow cytometry. RNA was extracted using an RNeasy Kit (Qiagen). Quality, concentration and integrity were verified by NanoDrop® ND1000 and inspection on formaldehyde-denaturing gels (28 S/18 S rRNA ratio ~2). The subsequent workflow (cDNA processing, Cy3-labelling, hybridization, washing, scanning) was carried out by Nimblegen (Roche, NZ). Briefly, cDNA was hybridized to a Nimblegen Mouse Gene Expression 12 × 135 k Array, containing 44,170 target genes with 3 × 60 mer replicate probe sets per target, based on the MM9 genome build. For mRNA-seq, the Poly(A) Purist™ MAG kit (Ambion) was used to extract mRNA from total RNA of the same six MEF samples submitted for microarray analysis. The quality was assessed using the Agilent 2100 Bioanalyzer. After submission of 250 ng of high-quality mRNA, the complete workflow (library preparation, quality control, sequencing) was carried out by NZ Genomics Ltd. (Dunedin, NZ). Libraries were generated using a TruSeq RNA Sample Prep Kit v2 (Illumina) and validated on a Bioanalyzer with a High Sensitivity DNA kit. Sequencing used the paired-end 2 × 100 protocol on an Illumina HiSeq. 2000.

### Microarray data analysis

Raw data were extracted using Nimblegen’s DEVA software. For each probe set, there was one perfect match (PM) and one mismatch (MM) probe, designed to have 100% identity and one mismatch, respectively, to the target sequence. With all MM probes being zero or near-zero, all PM probes were declared valid. Multi-array average normalization^61^ and background subtraction was performed using DEVA. Normalized data were re-loaded into R and examined on boxplots, revealing no outliers (outside 3 standard deviations of the mean). Contrasts for each probe and gene were done using the “limma” package in R.

### mRNA-seq data analysis

More than 90% of the bases had a Phred quality (Q) score >30, i.e. a base call accuracy of 99.9%. Flexbar software was used to remove low quality regions (<Q20) and sequencing adapters from the reads. This removed reads <18 bp after quality trimming and only kept properly paired reads. Each sample was mapped to the mouse reference genome (*Mus musculus* GRCm38 with NCBI annotations) using STAR version 2.5.0, resulting in ~62–85 Million (≈90%) mapped paired-end reads. The ~58–79 Million (≈85%) uniquely mapped paired-end reads for un-stranded mRNA-seq for each sample were compiled into a tab-delimited text file. Counts for each gene were analysed using the “edgeR” (version 3.10.5) package in R (version 3.2.1)^62^. Fold-changes between induced vs non-induced samples were calculated using a negative binomial model. Exact tests were used to calculate p-values, which were corrected for multiple testing using FDR-corrected q-values^63^. Fold-changes, p-values and FDR values were imported into Excel for further analysis. Significant up- and down-regulated genes were called using P < 0.05 and ≥1.5 fold-change as cut-offs.

To identify significantly deregulated repeat types, sequences of repetitive elements were downloaded from the Repeat Masker (RMSK) database (open-3-3-0 version of RMSK, RMSK library release 20120418). After sorting and converting the STAR (version STAR_2.5.0b) output with samtools v0.1.19-44428cd^64^, reads that mapped to repetitive elements were extracted using the feature Counts Version 1.5.0-p3^65^ using the “-p” option to count fragments instead of reads. Counts from individual units of the same repeat type were combined and total counts for each repeat type were calculated. Repeats with less than 10 fragments over all the samples were removed. Differentially expressed repeats were determined in edgeR. Count numbers for all repeats were used as input to estimate the effective library size and normalised using the trimmed mean of M method in edgeR. Fold-changes, p-values and FDR values for induced vs non-induced samples were calculated as above and called as significantly different at P < 0.05.

### Demethylase activity detection

A fluorometric assay (Cayman Chemicals, No. 700390) was analyzed on a multi-mode plate reader (Synergy 2, Biotek, USA) using an excitation/emission wavelength of 360/460 nm, respectively. Human recombinant KDM4B and H3K9me3 peptides provided positive controls.

### Immunofluorescence

Cells were simultaneously fixed and permeabilized in 4% (w/v) paraformaldehyde (PFA)/1% (w/v) Triton X-100 in PBS, blocked with 2.5% (w/v) bovine serum albumin in PBS and incubated overnight at 4–8 °C with primary antibodies^26^ (Table S9). Cells were washed in PBS-0.05% Tween^®^ and incubated for 1 h at room temperature with secondary antibodies. Cells were visualized with epifluorescence (AX-70 Olympus microscope). Images were processed using a Spot RT-KE slider CCD camera and software (Diagnostics Instruments Inc.). NT reconstructs were first fixed in 4% PFA and then permeabilized in 0.1% Triton X-100. Negative controls were processed the same way with blocking buffer instead of primary antibodies. For each reconstruct, the Hoechst-stained region of interest (ROI) was outlined in ImageJ. Mean grey value intensity, measured at 3 random cytoplasmic locations, was subtracted from the mean ROI intensity. This background-corrected mean intensity represents the sum of grey values of all pixels in the ROI divided by the number of pixels, and is referred to as normalized pixel intensity. Following quantification, images were pseudo-coloured using the cyan, green and red lookup tables.

### Western blot

Histones were extracted using the EpiQuik^TM^ Total histone extraction kit (Epigentek, Cat- OP-0006-100). Histone extracts (10–15 µg per lane) were separated on a 15% SDS PAGE gel, transferred onto a nitrocellulose membrane and probed with primary antibodies (Table S8). Following incubation with a secondary antibody, the modified histones were visualised with enhanced chemiluminescence. Signal intensity was normalized for the histone H1 signal (Ponceau S stain) and quantified using Quantity One software (Bio-Rad Laboratories Inc.).

### Nuclear transfer

NT donor cells were metaphase-arrested with 1.65 μM nocodazole for 2–3 hours, before shaking them off the plate^26^. Oocytes were collected from 10–12 weeks old B6C3 mice and the cumulus cells and zona pellucida removed. Zona-free NT was carried out as described^26^ but with two modifications. First, zona-free oocytes were enucleated at 30 °C under polarized light (CRi Oosight Imaging System). Second, shake-off MEFs were treated with 1 mg/ml pronase (in Hepes-buffered CZB medium) for 3 minutes prior to NT to improve fusion efficiency. NT reconstructs were kept in nocodazole until fusion had occurred. At 1 h post-fusion, reconstructs were activated with 10 mM SrCl_2_ in Ca-free M16. Only reconstructs with one polar body after 6 h were cultured in M16 drops at 37 °C under 5% CO_2_ in air. On day 4, compacted morulae and blastocysts were either processed for molecular analyses or transferred into the uterus of day 0.5 pseudo-pregnant females^26^. For some experiments, cells or embryos were treated with TSA or SCR at 50 nM and 250 nM, respectively, or respective DMSO dilutions (1:200) as controls. For NT, TSA and SCR were provided during activation (6 h) and early culture (3 h for TSA, 12 h for SCR).

### iPSC generation

Homozygous iPSC mice were imported from the Jackson laboratory (Stock no. 011004). This R26^*rtTA*^ strain expresses a Dox-inducible polycistronic four iPS factor (4 F) cassette, linked by self-cleaving 2 A peptides, from the Col1a1 locus (Col1a1^*4F2a*^). Mice were mated with either homozygous R26^*rtTA*^, Col1a1^*F*-*Kdm4b*^ or R26^*rtTA*^, Col1a1^*WT*^ controls. On day 12.5, MEFs were derived from both crosses. In addition to two *R26* ^*rtTA*^ alleles, these MEFs carry one copy of the Col1a1^*4F2a*^ transgene and either one copy of Col1a1^*F*-*Kdm4b*^ or one copy of Col1a1^*WT*^. Secondary iPSCs were derived from both MEF genotypes by culture in 2 µg/ml Dox. MEFs (passage 1–4) were seeded at ~1 × 10^4^ cells/cm^2^ in DMEM/F12 with Glutamax^TM^-I and 10% FBS. After three days, cells were passaged onto 0.1% gelatine-coated tissue culture dishes. After two days, cells were shifted into iPSC medium, comprising of PD0325901 (0.04 μM), GSK3B inhibitor CHIR99021 (3 μM), and recombinant human LIF (20 ng/ml) in DMEM/F12 supplemented with N2 and mixed 1:1 with Neurobasal medium supplemented with B27 and 1 mM L-glutamine (‘N2B27’)^66^. Culture medium was changed every 2–3 days. On D21, Dox was removed from the medium and the number of alkaline phosphatase- and NANOG-positive colonies counted on D28, i.e. one week after Dox removal. For detecting alkaline phosphatase activity, cells were fixed with 4% PFA and stained with NBT/BCIP reagent for 20 min. Dox-independent colonies were dissociated using 0.05% TrypLE™ and passaged every three days.

### Statistical analysis

Values are the average of several replicates (n) ± SEM, unless noted otherwise. Significance of differences for western analysis was determined via two-tailed paired t-tests on normalised band intensities. For the quantification of fluorescent signals and qPCR results, significance was determined by two-tailed t-tests. For comparing *in vitro* development, significance was determined using the two-tailed Fisher exact test for independence in 2 × 2 tables.

## Electronic supplementary material

Supplemental info

Table S1

Table S2
