# Supplementary material for: KDM4B-mediated reduction of H3K9me3 and H3K36me3 levels improves somatic cell reprogramming into pluripotency
Source: Sci Rep. 2017 Aug 8;7:7514. doi: 10.1038/s41598-017-06569-2 (PMC5548918; doi:10.1038/s41598-017-06569-2)
Supplement: Supplementary file 1 — Supplemental info [file 41598_2017_6569_MOESM1_ESM.pdf]

SUPPLEMENTARY INFORMATION

KDM4B-mediated reduction of H3K9me3 and H3K36me3 levels improves somatic cell reprogramming into pluripotency

Jingwei Wei, Jisha Antony, Fanli Meng, Paul MacLean, Rebekah Rhind, Götz Laible, Björn Oback

SUPPLEMENTAL MATERIALS AND METHODS

Teratomas

iPSC lines at passage 3-6 were harvested with a cell scraper, centrifuged and re-suspended in PBS + 1% PVA (10-30k). Using a 23G needle, 100 µl of cell suspension (approx. 1 - 5 x 10^6 cells per site) was injected intramuscularly into the quadriceps of adult immune-deficient mice. After 5–7 weeks, tumors were dissected, fixed overnight in Davidson's fixative, embedded in paraffin, sectioned, haematoxylin–eosin stained and analyzed by a pathologist service (NZVP, Hamilton, NZ).

Chimeras

iPSC lines were either aggregated with morulae or injected into host blastocysts (Swiss or FVB/NJArc) and transferred into the oviduct of day 0.5 pseudo-pregnant females. Coat-colour chimeras were bred with Swiss females for germline transmission.

SUPPLEMENTAL FIGURES

Figure S1

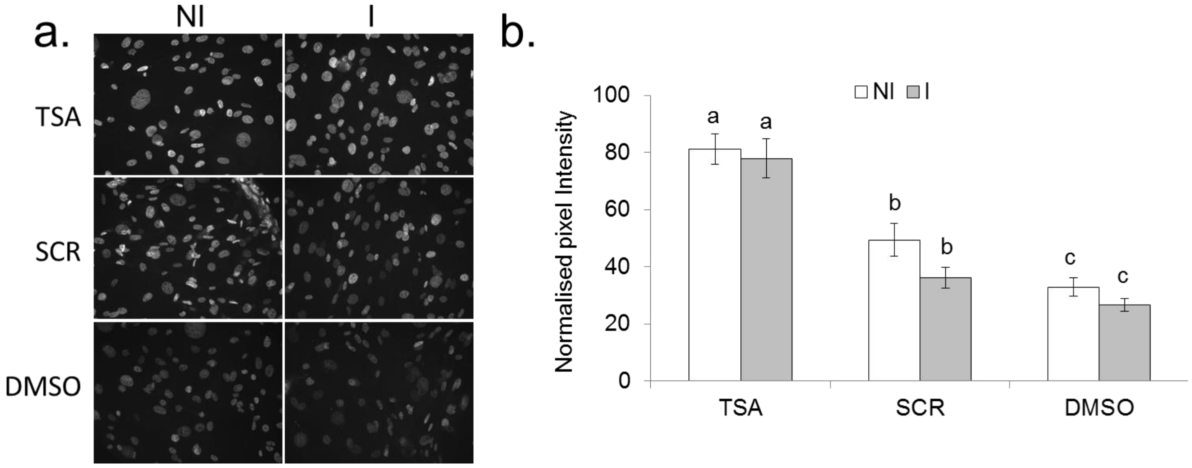

SUPPLEMENTAL FIGURE LEGENDS

Figure S1. Effect of HDACi on F-*Kdm4b*-EGFP expressing MEFs (a) Immunofluorescence of H3K9ac in 48 h non-induced (NI) vs induced (I) F-*Kdm4b* MEFs. **(b)** Quantification of immunofluorescence analysis in (A). Values represent normalized pixel intensity (ROI/area) ± SEM. Asterisks indicate significant differences between induced (I) vs non-induced (NI) MEFs and their corresponding HDACi treatments (N=20 nuclei quantified per treatment, n=4 replicates). Within each group (NI vs I), bars with different superscripts differ $^{ab}P<0.005$ by two-tailed unpaired t-test.

SUPPLEMENTAL TABLES

Supplemental Table 3. Functional annotation of differently expressed genes upon *Kdm4b*-induction

Top 3 Molecular and Cellular Functions*	***P-value***
Cellular Compromise	5.60E-03 - 1.65E-04
Cellular Function and Maintenance	5.60E-03 - 1.65E-04
Cell Death and Survival	3.74E-03 - 6.24E-04
Top 3 Canonical Pathways	***P-value***
Oncostatin M Signaling	2.10E-02
Inhibition of Matrix Metalloproteases	2.41E-02
Transcriptional Regulatory Network in Embryonic Stem Cells	2.47E-02
Top 3 Associated Network Functions	***Score***
Cell Cycle	27
Immunological Disease, Molecular Transport	3
Carbohydrate Metabolism, Skeletal and Muscular System Development and Function, Small Molecule Biochemistry	3

*Gene Ontology was determined by QIAGEN's Ingenuity® Pathway Analysis.

Supplemental Table 4: In vitro development of F-*Kdm4b* NT reconstructs treated ± HDACi

Induction[a]	HDACi[b]	n	nNT[c]	Morulae (% ± SEM)	Blastocysts (% ± SEM)
NI	-	5	130	1 (1 ± 0.2%)[a]	1 (1 ± 1%)[a]
I	-	12	230	16 (7 ± 1%)[b]	11 (5 ± 2%)
NI	SCR	2	59	4 (7 ± 1%)	9 (15 ± 2%)[b]
I	SCR	4	86	6 (7 ± 2%)	9 (11 ± 2%)
NI	TSA	3	81	4 (5 ± 1%)	3 (4 ± 1%)
I	TSA	9	155	12 (8 ± 1%)	7 (5 ± 1%)

[a]MEFs (#1, 3, 9) were either Dox-induced (I) or non-induced (NI) for 48 hours: [b]NT reconstructs were treated with scriptaid (SCR), trichostatin (TSA) or DMSO; [c]number of NT reconstructs that extruded a single polar body and were cultured until day 4. Significance was determined via Fisher 2x2 test; [ab]Cells with different superscripts within a column differ with P<0.01

Supplemental Table 5. Teratoma formation from *Kdm4b*-EGFP iPSC lines

Animal ID	Cell line	Dox	Tumor diameter (mm)	Ectoderm*	Mesoderm*	Endoderm*
1	#1 iPSC	_	20<br>20	√<br>ND	√<br>ND	√<br>ND
2	#1 iPSC	+	19<br>10	√<br>ND	√<br>ND	√<br>ND
3	#3 iPSC	_	No tumor	-	-	-
4	#7 iPSC	_	13	-	√	√
5	v6.5 ESC	_	15<br>13	√<br>ND	√<br>ND	√<br>ND

*Germ layer identity was determined on histological sections (Fig. 6D). ID=unique animal identifier; ND=not determined

Supplemental Table 6. Chimera formation from *Kdm4b*-EGFP iPSC lines

iPSC line	sex	No. of born mice	Chimaeras*	% of Chimaeras*
#1 Col1a1$^{4F2a/F\text{-}\mathit{Kdm4b}}$	♀	18	3	17%
#7 Col1a1$^{4F2a/F\text{-}\mathit{Kdm4b}}$	♂	2	1	50%

*Chimerism was determined by coat colour (Fig. 6E).

Supplemental Table 7: Primer sequences used for qPCR

**Primer**		**Sequence (5'- 3')**	**Size (bp)**	**Reference**
2810474019Rik	F:	TTTCTGTCTCGGTCTTCCGC	181	This paper
	R:	CACGCCTCTAATTCTGTTTTTCCA		
Actb	F:	CAGAAGGACTCCTATGTGGG	200	[1]
	R:	TTGGCCTTAGGGTTCAGGG		
Transgene Kdm4b	F:	AGAAGACACCGGGACCGATC	190	[2]
	R:	TGAATTCATCCATGGTGGGG		
C430002N11Rik	F:	AGCCCCTTCGGCTGTTTTTA	136	This paper
	R:	GGGCTGAACCTCCTTCTGTC		
Chst13	F:	ATGGGAAGACGCTCCTGTTG	98	This paper
	R:	TTTTCAAATGCGGGACGCAG		
Cola1 (Wild-type)	F:	GCTCGCACGTACTTCATTCC	400	This paper
	R:	GAGAGTTCCTTGAGGGCTGG		
Endo Kdm4B	F:	AGAAGCCTTCCTGTTCTCAG	189	[2]
	R:	TGTACTGACTGGCTGTAGGG		
Ephx2	F:	GGGAAAGGAATTTACCCCGCT	72	This paper
	R:	GAGTGGTACCCACTGGGCTG		
Gapdh	F:	TGCACCACCAACTGCTTAG	176	[1]
	R:	GATGCAGGGATGATGTTC		
Glipr1	F:	CGTGCAGTGATTGCCCAAAA	90	This paper
	R:	CGACAGAGTAGTAACGTGAGACC		
Gpr56	F:	TTTTCTCTGGTGCAAGGTGC	192	This paper
	R:	GAAGCGGGGAATATCTGGGG		
Gusb	F:	ATAAGACGCATCAGAAGCCG	96	[3]
	R:	ACTCCTCACTGAACATGCGA		
Hprt	F:	GAAATGTCAGTTGCTGCGTC	332	[4]
	R:	GCCAACACTGCTGAAACATG		
Lrrn1	F:	TCCTCATCCTCCGGCTAGTG	93	This paper
	R:	GAGACGTGTCCTAAGGCTGG		
Ly6a	F:	GGAGCTGCTAGGTTTTATCTGTGC	178	This paper

	R:	GGGCAGGTAATTGATGGGCA		
M2-rtTA	F:	GAGAGTTCCTTGAGGGCTGG	407	This paper
	R:	CAATTGCTTGTCTCAGAAGTGG		
Mmp13	F:	GGAGCCCTGATGTTTCCCAT	108	This paper
	R:	GTCTTCATCGCCTGGACCATA		
Nnat	F:	CCACCCACTTTCGGAACCAT	104	This paper
	R:	ACTGGGGACAGGGTCTGC		
Prss42	F:	GAAGAGGGGAAGTGGCCTTG	130	This paper
	R:	CGTTGTACTGGATTCGGCTGTA		
Rosa26 (Wild-type)	F:	GCTGTTTTGGAGGCAGGAAG	700	This paper
	R:	TGCCAATGCTCTGTCTAGGG		
Serpina3g	F:	ACAGAGGCTGAAAAGGAGCAG	139	This paper
	R:	AGAGGCTGAAGGCAAAGTCA		
Slc29a3	F:	CTTTGAGAGCTACCTGGCAGT	91	This paper
	R:	GTGCACCTGGACCCTGTTGA		
Sry	F:	TGGGACTGGTGACAATTGTC	402	[5]
	R:	GAGTACAGGTGTGCACCTCT		
Trim24	F:	GCCATTCGCCACCCAAGT	150	This paper
	R:	CAGCTTGTACATACCTGATTAGACT		
Zfp37	F:	GGACCTGACAAAGCCAGAGG	198	This paper
	R:	TGATTTCCCATGGAGGCTGG		

Supplemental Table 8: Overview of MEF lines used for the various assays

**Assay**	**Induction**	**MEF lines**															
		F-Kdm4b							**M-*Kdm4b***	***F-Kdm4b/iPS***			***M-Kdm4b/iPS***		***WT/iPS***
	Dox (days)	#1 (♂)	#3 (♀)	#4 (♀)	#5 (♀)	#6 (♀)	#9 (♂)	#11 (♀)	#13 (♀)	#1 (♀)	#2 (♂)	#7 (♂)	#1 (♀)	#3 (♀)	#1 (♂)
Flow cytometry	1-2	√	-	√	√	√	√	√	√	-	-	-	-	-	-
qPCR	2	-	-	√	√	√	-	√	-	-	-	-	-	-	-
Microarray	2	-	-	√	√	√	-	-	-	-	-	-	-	-	-
mRNA-seq	2	-	-	√	√	√	-	-	-	-	-	-	-	-	-
Immunofluorescence	2	√	√	√	√	-	-	√	-	-	-	-	-	-	-
Western blot	2	-	-	-	√	-	-	√	-	-	-	-	-	-	-
Demethylase activity	2	-	-	-	√	√	-	-	-	-	-	-	-	-	-
NT	1	-	-	√	√	√	-	√	√	-	-	-	-	-	-
NT ± HDACi	1	√	√	-	-	-	√	-	-	-	-	-	-	-	-
iPS colony formation	21	-	-	-	-	-	-	-	-	√	√	√	√	√	√
Teratoma or chimera	21	-	-	-	-	-	-	-	-	√	√	√	-	-	-

√=tested for this assay; -=not determined; NT=Nuclear transfer, WT=wild-type

Supplemental Table 9: Primary and secondary antibodies

Antibody	*Dilution*		*Manufacturer (Cat.-no.)*
	IF	WB	
Alexa Fluor donkey anti mouse 488	1:1000	NA	Thermo Fisher (Cat-A11008)
Alexa Fluor goat anti rabbit 568	1:1000	NA	Thermo Fisher (Cat-A11011)
Goat anti rabbit HRP	NA	1:10000	Thermo Fisher (Cat-G21234)
Mouse anti GFP	1:1000	NA	Thermo Fisher (Cat-A11120)
Rabbit anti H3K9Ac	1:250	NA	Millipore (Cat-04-1003)
Rabbit anti H3K9me1	1:1000	1:6000	Gift by T Jenuwein (Freiburg, Germany)
Rabbit anti H3K9me2	1:1000	1:3000	Gift by T Jenuwein (Freiburg, Germany)
Rabbit anti H3K9me3	1:1000	1:3000	Gift by T Jenuwein (Freiburg, Germany)
Rabbit anti H3K27me3	1:1000	1:3000	Gift by T Jenuwein (Freiburg, Germany)
Rabbit anti H3K36me3	1:500	1:1000	Cell Signaling (#9763)
Rabbit anti NANOG	1:100	NA	Abcam (ab80892)

NA=not applied

SUPPLMENTAL REFERENCES

1 Pearton, D. J. *et al.* Elf5 counteracts precocious trophoblast differentiation by maintaining Sox2 and 3 and inhibiting Hand1 expression. *Dev Biol* **392**, 344-357, doi:10.1016/j.ydbio.2014.05.012 (2014).

2 Antony, J., Oback, F., Chamley, L. W., Oback, B. & Laible, G. Transient JMJD2B-Mediated Reduction of H3K9me3 Levels Improves Reprogramming of Embryonic Stem Cells into Cloned Embryos. *Molecular and cellular biology* **33**, 974-983, doi:10.1128/MCB.01014-12 (2013).

3 Tiedt, R. *et al.* Ratio of mutant JAK2-V617F to wild-type Jak2 determines the MPD phenotypes in transgenic mice. *Blood* **111**, 3931-3940, doi:10.1182/blood-2007-08-107748 (2008).

4 Peters, J. *et al.* A cluster of oppositely imprinted transcripts at the Gnas locus in the distal imprinting region of mouse chromosome 2. *Proceedings of the National Academy of Sciences* **96**, 3830-3835, doi:10.1073/pnas.96.7.3830 (1999).

5 Lambert, J.-F. *et al.* Quick sex determination of mouse fetuses. *Journal of Neuroscience Methods* **95**, 127-132, doi:10.1016/s0165-0270(99)00157-0 (2000).
